# Genomic Footprints in Selected and Unselected Beef Cattle Breeds in Korea

**DOI:** 10.1371/journal.pone.0151324

**Published:** 2016-03-29

**Authors:** Dajeong Lim, Eva M. Strucken, Bong Hwan Choi, Han Ha Chai, Yong Min Cho, Gul Won Jang, Tae-Hun Kim, Cedric Gondro, Seung Hwan Lee

**Affiliations:** 1 Division of Animal Genomics & Bioinformatics, National Institute of Animal Science, RDA, Jeonju 565–851, Republic of Korea; 2 School of Environmental and Rural Science, University of New England, Armidale, NSW Australia; 3 Division of Animal and Dairy Science, Chung Nam National University, Daejeon 305–764, Republic of Korea; Humboldt-University Berlin, GERMANY

## Abstract

Korean Hanwoo cattle have been subjected to intensive artificial selection over the past four decades to improve meat production traits. Another three cattle varieties very closely related to Hanwoo reside in Korea (Jeju Black and Brindle) and in China (Yanbian). These breeds have not been part of a breeding scheme to improve production traits. Here, we compare the selected Hanwoo against these similar but presumed to be unselected populations to identify genomic regions that have been under recent selection pressure due to the breeding program. *Rsb* statistics were used to contrast the genomes of Hanwoo versus a pooled sample of the three unselected population (UN). We identified 37 significant SNPs (FDR corrected) in the HW/UN comparison and 21 known protein coding genes were within 1 MB to the identified SNPs. These genes were previously reported to affect traits important for meat production (14 genes), reproduction including mammary gland development (3 genes), coat color (2 genes), and genes affecting behavioral traits in a broader sense (2 genes). We subsequently sequenced (Illumina HiSeq 2000 platform) 10 individuals of the brown Hanwoo and the Chinese Yanbian to identify SNPs within the candidate genomic regions. Based on allele frequency differences, haplotype structures, and literature research, we singled out one non-synonymous SNP in the *APP* gene (*APP*: c.569C>T, Ala199Val) and predicted the mutational effect on the protein structure. We found that protein-protein interactions might be impaired due to increased exposed hydrophobic surfaces of the mutated protein. The *APP* gene has also been reported to affect meat tenderness in pigs and obesity in humans. Meat tenderness has been linked to intramuscular fat content, which is one of the main breeding goals for brown Hanwoo, potentially supporting a causal influence of the herein described nsSNP in the *APP* gene.

## Introduction

Hanwoo is an indigenous cattle breed of Korea that has been maintained with minimal introduction of other germplasm for >2,000 years **[1].** The brown Hanwoo have been subjected to intensive artificial selection over the past four decades to improve meat production traits such as carcass weight, eye muscle area, marbling (intramuscular fat), and meat tenderness **[2, 3]**. This artificial selection has potentially increased the frequency of favorable alleles in brown Hanwoo (HW) at loci affecting the production traits. Nowadays, HW is one of the top breeds for intramuscular fat next to Japanese Wagyu cattle. Outside of the breeding program for HW, two closely related but unselected varieties survived in Korea: Brindle Hanwoo (BR) and Jeju Black Hanwoo (JB) **[4, 5]**. These two strains have small populations with around 4,000 BR individuals and 1,000 JB. Each population is primarily found on two islands off the coast of Korea and might be considered as island populations in which a founder effect and genetic drift contributed to a loss of variation and differentiation from the mainland HW. Chinese Yanbian (YB) cattle are found in the Yanbian Prefecture in China, north of the border of North Korea. The region has a strong Korean influence (60% of the population were Korean until the 1950s) and the Yanbian cattle were potentially fully connected to the brown Hanwoo until the split between North and South Korea. Yanbian are mainly used as draft animals and remained unselected for production traits **[6].**

There are several population genetic occurrences that will shift allele frequencies such as migration and admixture, bottle-necks, or random genetic drift. However, only constant inbreeding or selection will increase homozygosity in a population in a short amount of time. Whilst inbreeding will affect the entire genome and is tried to be minimized in modern selection programs, selection will accumulate favorable alleles targeting specific genetic regions. Genome scans for such signatures of selection in single breeds have identified known candidate genes such as *DGAT1*, the casein cluster, or *GHR* **[7–9]**. Applied statistics for a single breed include *site frequency spectrum* (*SFS*) or *extended haplotype homozygosities* (*EHH*) **[10–12]**. A more powerful approach is to compare genomes of populations that have been under different selection pressures. Such comparison studies were successfully carried out between dairy and beef breeds **[13, 14]**, new world, European, and African cattle breeds **[15],** or within East-Asian cattle breeds **[16].** Comparison statistics include *F*_*ST*_, *XPEHH*, or *Rsb* which are derived from the *EHH* statistic **[17–19]**. All of these statistics were designed to assess the similarity or difference of two populations based on genomic information.

We decided to compare the genomes of the selected HW with the unselected breeds of BR, JB, and YB to narrow down genetic regions with accumulated homozygosity in the HW. Such regions might have been shaped by the intensive artificial selection in the HW, and therefore, could be hotspots for genes affecting the traits emphasized in the Korean breeding program. We investigated the genes with known effects that are located in the identified selective sweep regions to provide explanations to why these regions might be under selection pressure in the HW. Lastly, we sequenced the entire genome of selected HW and YB individuals to obtain a more precise picture of sequence variations in regions of interest that might not have been captured by genome-wide markers. The DNA sequence was further used to predict protein structures and the impact of found sequence variations on protein structure.

## Results and Discussion

### Population structure and diversity

**Fig 1:**
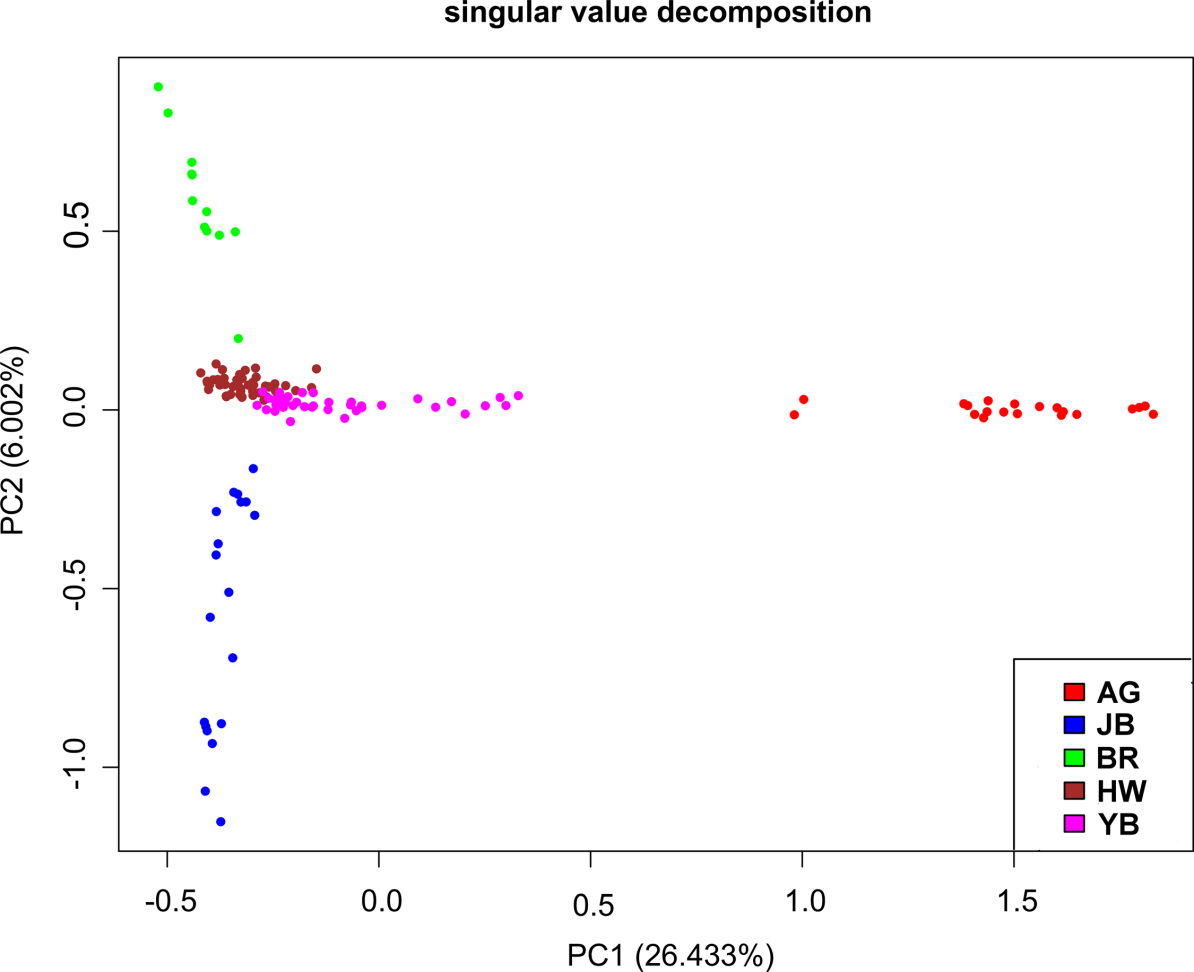
First and second principal components based on the genomic relationship matrix of 5 cattle breeds. The first component separates the European Angus from the East-Asian cattle breeds and explains 26.4% of the variation amongst the individuals. The second component separates the East-Asian breeds and explains 6% of the variation. HW: Hanwoo; BR: Brindle Hanwoo; JB: Jeju Black Hanwoo; YB: Chinese Yanbian; AG: Angus.

**Fig 2:**
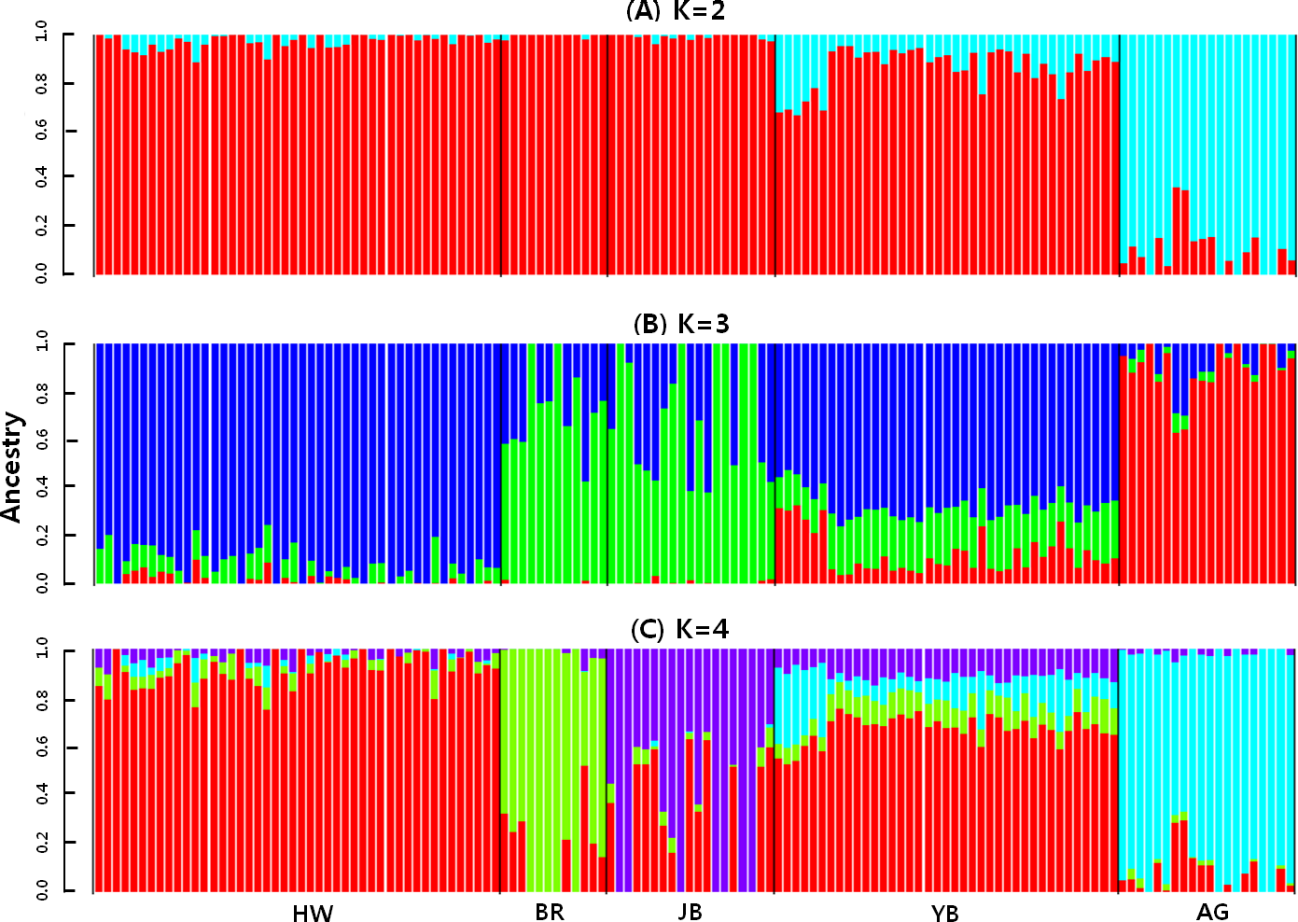
Unsupervised hierarchical clustering of individuals genotyped for 38,266 SNP for K = 2, 3 and 4 (best fit K = 3). Each color depicts the breed proportions per individual and based on allele frequencies of the breeds available for this study. HW: Hanwoo; BR: Brindle Hanwoo; JB: Jeju Black Hanwoo; YB: Chinese Yanbian; AG: Angus.

We performed a principal component (PCA) and Admixture analysis based on genotypic information which provided information about the genetic relationship of breeds and individuals within the breeds. The average genomic relationship between animals within the current population was 0.003, 0.049, 0.05 and 0.037 for HW, BR, JB, and YB, respectively (**S1 Fig**). In addition to the four East-Asian breeds, we included a European taurine breed (Angus) in the analysis to provide an anchor point for the breed diversification. The PCA results grouped individuals of the four East-Asian and the Angus breed in agreement to their origins **(Figs 1 and 2).** Some of the YB cattle spread from the East-Asian cluster to the out-group of Angus cattle. This could suggest that YB either had some unaccounted intercrossing with European cattle breeds, or genetic artefacts of the same domestication origin as European taurine remain in this unselected population **[20]**. Further, HW and YB cattle are genetically very similar whilst BR and JB are more distinguishable (**Table 1, Fig 2**), probably due to the more intense drift and founder effects in these small populations. Nevertheless, compared to other breed differences, these two cattle varieties can be classified as Hanwoo.

**Table 1:** Pairwise F_ST_ (lower diagonal) and genomic relationships (upper diagonal) of 5 cattle breeds.

	AG	JB	BR	HW	YB
**AG**	-	0.032132	0.035135	0.028352	0.022256
**JB**	0.167763	-	0.017421	0.010771	0.008228
**BR**	0.166517	0.001246	-	0.01149	0.009939
**HW**	0.23765	0.069886	0.071132	-	0.004175
**YB**	0.253095	0.085332	0.086578	0.015445	-

AG: Angus; JB: Jeju Black Hanwoo; BR: Brindle Hanwoo; HW: Brown Hanwoo; YB: Chinese Yanbian

Based on Admixture results, the BR and JB cattle have on average 30% of their allelic frequencies in common with HW, and YB share on average 67.2% of their genetic make-up with HW (**Fig 2B**). As a comparison, the East-Asian cattle breeds shared only between 0.2% and 1.5% of their genetic make-up with the European Angus cattle. Further, *F*_*ST*_-statistics, which is designed to describe differences between two populations based on allele frequencies, also found that the Angus cattle were more distantly related to the Korean breeds than they are amongst each other (**Table 1**). Closest relationships were found between the YB and the other Korean breeds, especially with HW. Lastly, genomic relationships within the populations were <0.01 for the East-Asian breeds, indicating low relatedness and random sampling of animals. The similarity of the selected East-Asian cattle breeds makes them a good choice to detect recent selection footprints in the brown Hanwoo caused by the relatively recent breeding program.

### Identification of footprints of selection

**Fig 3:**
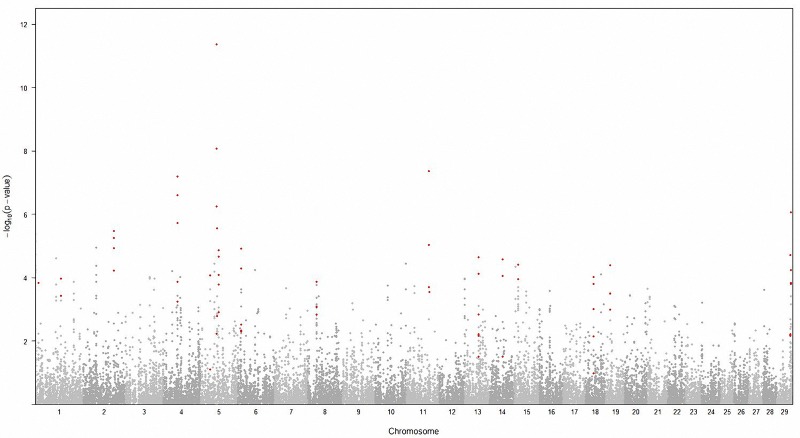
Genomic regions with selection signals in the Brown Hanwoo vs. a pooled unselected population using *Rsb* statistic. Red dots indicate significant markers remaining after FDR correction.

Footprints of selection were analyzed with the *Rsb* statistic which is based on *extended haplotype homozygosities* (*EHH*). *Rsb* scores are designed to detect recent selective sweep regions that occur in only one population compared to another. We compared the HW against a pooled sample of unselected populations (UN including the BR, JB, and YB) to increase the population and create a better contrast. All chromosomes displayed regions with significant *P*_*Rsb*_ scores (*P*_*Rsb*_ = -log[Φ(*Rsb*)]) indicating HW specific sweep regions (**fi**). To narrow down the regions of interest, we created 5,103 windows across the genome covering 1 Mb each (average 14.96 SNP per window). Candidate regions were considered those windows containing at least two SNP with *P*_*Rsb*_ > 2 (P-value < 0.01). At this stage, 31 suggestive regions containing 185 SNPs were identified (**S1 Table**). We further applied the Benjamini-Hochberg false discovery rate (FDR P-value < 0.05) to correct for multiple testing (**Fig 3, [21]**). Out of the 31 suggestive regions, 16 candidate regions containing 37 SNPs remained significant (**Table 2**).

**Table 2:** Genomic regions with significant evidence (FDR corrected, selected *versus* unselected populations) for recent positive selection in brown Hanwoo based on the *Rsb* statistic.

Candidate regions (Chr, Mb)		Significant SNPs (P-value)	Genes in: region*	Trait associations	QTL information
chr1	9–10.5	Hapmap38887-BTA-22281 (0.038)	*APP*	meat tenderness, obesity [23–25]	weaning weight-maternal milk
			*GABPA*	obesity [25]	
chr1	85–86.5	BTB-01702174 (0.047)	*-*	-	marbling score, Longissimus muscle area,
chr2	102.5–104	ARS-BFGL-NGS-3990 (0.006), ARS-BFGL-NGS-107381 (0.034), Hapmap34329-BES11_Contig247_1378 (0.014), ARS-BFGL-NGS-67961 (0.01)	*ACADL*	fatty acids oxidation [70]	-
			*MYL1*	skeletal muscle differentiation [71]	
chr4	47–48.5	BTB-01351245 (0.049), BTB-01351221 (0.001), ARS-BFGL-NGS-38285 (0.0003), ARS-BFGL-NGS-108242 (0.004)	*RELN*	neural development [33]	body weight, body energy content, body length, length of productive life
chr5	32.5–34	ARS-USMARC-635 (0.041)	*WNT1*	coat color, melanocyte expansion [31]	body weight (slaughter, birth), Average daily gain, Subcutaneous fat increase
			*FAIM2*	obesity [72]	
chr5	54.5–56	BFGL-NGS-111500 (0.002), Hapmap42656-BTA-73551 (0.00008), BTA-73549-no-rs (0.0000001), BFGL-NGS-111906 (0.006)	*AVPR1A*	mammalian social behaviour [34]	body weight (slaughter, birth), body size, height
chr5	61–62.5	Hapmap34521-BES10_Contig695_990 (0.041), Hapmap34864-BES11_Contig325_836 (0.02), ARS-BFGL-NGS-44271 (0.016)	*CD63*		body weight, pre-weaning average daily gain
			*MYL6A*		
			*MYL6B*	skeletal muscle generation [73]	
chr6	10–11.5	Hapmap42479-BTA-22862 (0.015) BTA-88441-no-rs (0.033)	*NDST4*	fatness traits [74]	body weight(birth, weaning, slaughter), Average daily gain
chr8	28.5–30.5	BTB-01271445 (0.049)	*SH3GL2*	lipid binding and diabetis [75]	average daily gain, residual feed intake, body weight
chr11	75.5–77	Hapmap35883-POMC_121F2-SNP1 (0.0002), ARS-BFGL-NGS-39507 (0.013)	*POMC*	obesity and coat color [32]	body weight (mature, weaning)
chr13	45.5–47	BFGL-NGS-116062 (0.039), BFGL-NGS-116977 (0.02)	*ADRB2*	body mass index and adipogenesis [76]	feed conversion ratio
chr14	41.5–43	ARS-BFGL-NGS-59210 (0.022), BTB-01709715 (0.043)	*FABP4*	intramuscular fat [77]	average daily gain, body weight(birth)
			*FABP5*		
chr15	12.5–14.5	Hapmap35322-BES8_Contig457_1759 (0.028), ARS-BFGL-NGS-17747 (0.047)	*MTMR2*	mammary gland metabolism [28]	body weight(birth, yearling), semen volume
			*CWC15*	reproduction [29]	
chr18	25.5–27	UA-IFASA-4931 (0.049), ARS-BFGL-BAC-34198 (0.045)	*-*	-	resigual feed intake, body weight (weaning)
chr19	15–16.5	UA-IFASA-8305 (0.028)	*ACCN1*	QTL for reproduction [30]	calf size, body weight (mature, yearling), residual feed intake
chr29	44.5–47	CAPN1_1 (0.02), BTA-66033-no-rs (0.034), ARS-BFGL-NGS-74975 (0.002), ARS-BFGL-NGS-20927 (0.049), ARS-BFGL-NGS-34609 (0.049)	*CAPN1*	carcass trait and meat tenderness [78, 79]	body capacity, Tenderness score, Shear force, resudial feed intake

*APP, Amyloid Beta (A4) Precursor Protein; GABPA, GA-Binding Protein alpha; ACADL, Acyl-CoA dehydrogenase, long chain; MYL1, Myosin, Light Chain 1; RELN, Reelin; WNT1, Wingless-Type MMTV Integration Site Family, Member 1; FAIM2, Fas Apoptotic Inhibitory Molecule 2; AVPR1A, Arginine Vasopressin Receptor 1A; CD63, CD63 Molecule; MYL6A, Myosin Light Polypeptide 6 alpha; MYL6B, Myosin Light Polypeptide 6 beta; NDST4, N-Deacetylase/N-Sulfotransferase (Heparan Glucosaminyl) 4; SH3GL2, SH3-Domain GRB2-Like 2; POMC, Proopiomelanocortin; ADRB2, Adrenoceptor Beta 2; FABP4, Fatty Acid Binding Protein 4; FABP5, Fatty Acid Binding Protein 5; MTMR2, Myotubularin Related Protein 2; CWC15, CWC15 Spliceosome-Associated Protein; ACCN1, Amiloride-sensitive Cation Channel 1; CAPN1, Calpain 1

All but one significant candidate region overlapped with QTLs for meat and production traits according to a search in the Animal QTLdb (Release 17; http://www.animalgenome.org/cgi-bin/QTLdb/BT/index). Among the 16 significant candidate regions, 21 known protein coding genes were located 300kb up-or down-stream of the SNP. Most of these genes (14 genes) are known to affect meat production, obesity, or lipid metabolism such as *GHRH* [22], *APP* **[23–25]**, or *TRPC1* **[26].**

The brown Hanwoo cattle have been selected for meat production since the 1970s **[2].** The breeding goal heavily emphasizes traits such as marbling score and meat tenderness, carcass weight, backfat thickness, and eye muscle area **[27]**. Over the past 30 years, the annual genetic gain in HW increased from 0.02 to 0.82 kg/year, with body weight (at 18 months of age) increasing from 331 to 574 kg, and intramuscular fat (final slaughter age) from 15% to 23% (**NIAS, 2009**). Therefore, we can assume that changes to the genetic make-up of HW cattle in favor of meat enhancing alleles had taken place which was supported by the large number of QTLs and genes affecting meat traits within the identified candidate regions **(Table 2)**.

Other genes affected reproduction including mammary gland development (3 genes: *MTMR2* **[28]**, *CWC15* **[29]**, *ACCN1* **[30]**), coat color (2 genes: *WNT1* **[31]**, *POMC* **[32]**), and behavioral traits, including autism, neural development defects, and maternal behavior in humans, and aggressive behavior in pigs (2 genes: *RELN* **[33]**, *AVPR1A* [34–36], **Table 2).**

Selection footprints in regions of fertility and mammary gland traits can be explained by the need of any breeding scheme to produce healthy and fertile individuals that are capable of nurturing their offspring to produce sufficient replacement animals **(Table 2, [37])**. Additionally, calm and social animals are easier to handle and keep under constricted housing conditions **[38, 39],** which is why the behavioral genes might have been indirectly selected for in the HW **(Table 2)**. The coat color genes were most likely identified due to the variation in this trait between the chosen populations **(Table 2).**

Only one of our identified regions (chr. 29 44.5-47Mb) was in agreement with a previous study on selection signatures in Korean beef cattle **[16]**. This region contained the *calpain 1* (*CAPN1*) gene which was reported to be associated with carcass weight and marbling score in Hanwoo [40]. This lack of confirmation with previous studies might be due to a sampling bias between the studies or the nature of the comparison in this study. This study was designed to identify recent signatures of selection **[27]**, rather than older signatures most likely due to natural selection (e.g. adaptive signatures for different environments) as reported in Porto-Neto et al. **[41]**.

### Detection of sequence variation in the selection signals

To provide further information and proof for the regions exhibiting positive selection footprints in HW, we sequenced the entire genome of the HW and YB populations (10 individuals of each group). The sequence data consisted of a total of 7,268,700,964 sequence-reads across the bovine genome. The average depth of sequence coverage for each sample was approximately 11-fold. The GATK and ANNOVAR analyses identified 103 SNP (53 nsSNP, 50 synonymous SNP) in the 16 candidate regions described in the previous section. Allele frequencies of the same allele in the HW and YB population were calculated for each nsSNP. Frequencies differed by more than 20% between the two populations for 23 nsSNP, however, significant differences were only found for 6 nsSNPs (**Table 3**). Out of these 6 nsSNP, 3 nsSNP were classified by PolyPhen as benign, 2 nsSNP as possibly damaging, and 1 nsSNP as probably damaging (**Table 3**).

**Table 3:** Allele frequencies and mutation effects for non-synonymous SNPs in known genes for brown Hanwoo (HW) and Chinese Yanbian (YB) cattle.

Candidate region		Gene Symbol	REF/ALT^a^	Amino acid	Allele Frequency of ALT^c^		PolyPhen^d^
(Chr, Mb)		(Ensembl ID)	allele	Substitution^b^	YB	HW	
chr1	9–10.5	*APP*	C/**T**	Ala/Val	0.6	0.9 **	possibly damaging
chr29	44.5–47	*SNX32*	G/**A**	Gly/Arg	0	0.45**	possibly damaging
chr5	32.5–34	*FAM186A*	G/**A**	Gly/Arg	0.65	1**	probably damaging
chr5	32.5–34	*FAM186A*	T/**G**	Ile/Met	0.1	0.3	probably damaging
chr29	44.5–47	*CDCA5*	C/**T**	Pro/Leu	0.7	0.95†	probably damaging
chr1	85–86.5	*B3GNT5*	C/**G**	Asp/Glu	0	0.2	benign
chr1	85–86.5	*YEATS2*	C/**T**	Pro/Ser	0.8	1	benign
chr1	85–86.5	*MCF2L2*	A/**T**	Lys/Met	0.75	0.95	benign
chr5	32.5–34	*FAIM2*	C/**T**	Ala/Val	0.7	0.95†	benign
chr5	32.5–34	*FAM186A*	G/**A**	Ala/Thr	0.6	0.8	benign
chr5	32.5–34	*FAM186A*	A/**G**	Lys/Glu	0.1	0.3	benign
chr8	28.5–30.5	*CNTLN*	T/**G**	Met/Arg	0.5	0.7	benign
chr13	45.5–47	*IDI1*	G/**A**	Gly/Glu	0.6	0.9†	benign
chr14	41.5–43	*FABP9*	C/**G**	Asp/Glu	0.35	0.65	benign
chr14	41.5–43	*FABP9*	G/**A**	Cys/Tyr	0.35	0.55	benign
chr15	12.5–14.5	*ENDOD1*	A/**C**	Glu/Asp	0.1	0.4†	benign
chr29	44.5–47	*CDCA5*	C/**G**	Asp/Glu	0.1	0.55**	benign
chr29	44.5–47	*MUS81*	A/**G**	Ile/Val	0	0.4**	benign
chr29	44.5–47	*SLC25A45*	A/**G**	Arg/Gly	0.2	0.55†	benign
chr29	44.5–47	*FRMD8*	T/**C**	Trp/Arg	0.55	0.9*	benign
chr29	44.5–47	*SCYL1*	C/**T**	Leu/Phe	0.5	0.75	benign
chr29	44.5–47	*POLA2*	A/**G**	His/Arg	0.2	0.4	benign
chr29	44.5–47	*EHBP1L1*	T/**C**	Val/Ala	0.8	1	benign

^a^ Base pairs of the nsSNP.

^b^ Amino acid substitutions predicted from reference genes (Ensembl ID) using ANNOVAR program.

^c^ The p-value of the difference of allele-frequency in HW and YB population based on Fisher's exact test are shown as

† P<0.1

*P<0.05

**P<0.01

***P<0.001.

^d^ PolyPhen classification of mutation effect on protein structure.

Out of the 3 possibly or probably damaging nsSNP, i.e. protein changing, only one gene matched putative candidate genes that were found within the selective sweep regions described in **Table 2**: The *amyloid beta (A4) precursor protein* (*APP*) on chromosome 1 (9–10.5 Mb). The *APP* gene was previously associated with meat and fat traits in pigs and mice **[42–44].** Further, analyses of haplotype structures around the five possibly or probably damaging nsSNP showed a haplotype block in the HW population only in proximity to the *APP* gene (located between markers ARS-BFGL-NGS-43310 and Hapmap38887-BTA-22281, **S2 Fig).** In theory, the linkage disequilibrium between markers weakens over time. However, artificial selection accumulates the favorable allele including a small region surrounding the mutation which manifests itself as a haplotype block. Therefore, we decided to investigate the *APP* gene in more detail.

The nsSNP in exon 5 of the *APP* gene (APP: c.569C>T, Ala199Val) had a 30% difference in allele frequencies between the HW and YB populations (p-value = 0.002, **Table 3**). The frequency of the Valine variant (minor T allele) was increased in the HW population compared to the YB indicating an accumulation of a possibly favorable variant under selection pressure.

The *APP* is a highly conserved gene and its protein is found in a variety of tissues as a cell surface receptor and transmembrane precursor. Several different splice variants are known **[45–49]**; however, the exact function of the protein remains unclear. The human APP protein consists of five domains including a transmembrane domain linking the ecto- and cytoplasm. Dawkins and Small **[50]** discuss several possible functions of the APP protein from trophic actions including cellular growth and neuronal recovery, to stem-cell proliferation, to blood coagulation. The *APP* gene is possibly best known for its involvement in Alzheimer’s disease as its protein is a precursor molecule for beta-amyloid (Aβ) which is a major component of amyloid plaques **[51]**. Further, *APP* has also been associated with a common progressive muscle disease (inclusion body myopathy, IBM) in elderly people **[52, 53].** Lobjois et al. **[42]** inferred that this progressive muscle disease might explain a link to meat tenderness in livestock populations. Currently, 40 coding mutations in the human *APP* gene are known; however, for exon 5 only one mutation was reported without a pathogenic effect **[54, 55].**

**Fig 4:**
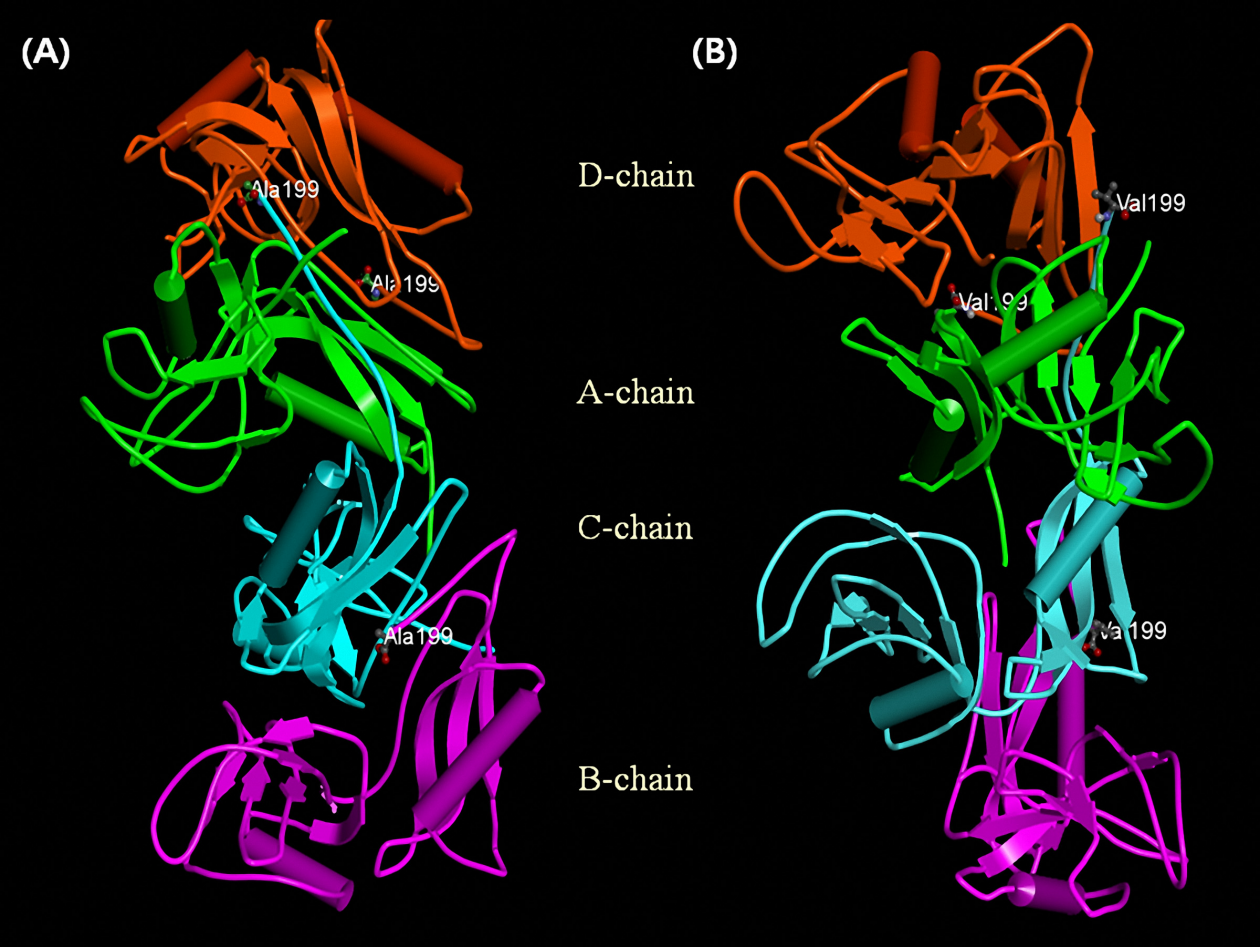
**The schematic structure of bovine *APP* gene in the wild-type (A) and mutated form (B).** The bovine protein structure and mutation effects were modelled according to the known template structure of the human gene (PDB code 3KTM). Mutation residue (Ala199Val) is represented by ball and stick.

We modelled the structure of the bovine APP protein based on the entire human N-terminal APP-E1 domain with a sequence identity to the bovine sequence of 92%. Only four out of the five known subunits of the human APP protein were also predicted in the bovine protein structure (**Fig 4, [50]**). A Kunitz-type protease inhibitor domain that was reported for longer isoforms of the human APP was missing. The position of the Ala199Val amino acid exchange in exon 5 was predicted to result in exposed hydrophobic surfaces of parts of the protein interfaces. Such an exposition is energetically unfavorable especially in aqueous environments. The folding free energy of each structure is defined as the free energy difference between the folded and unfolded states of the protein. Further, the folding structure of three out of the four protein subunits was affected by this nsSNP resulting in predicted unfavorable secondary and tertiary folding energies **(Table 4).** The pathogenic effect of the *APP* gene in humans was linked to an overexpression and thus an accumulation of Aβ peptides in brain and muscle cells **[52]**. Lobjois et al. **[42]** also reported that meat of pigs with a lower shear force had a higher expression of APP in the longissimus dorsi muscle. Possibly, our identified nsSNP could cause an accumulation of Aβ peptide in the muscle through an impaired protein-protein interaction which could inhibit post-translational protein cleavage, and thus, affect meat tenderness.

**Table 4:** The predicted mutation effect on the protein structure stability of the bovine *APP*:c.569C>T, Ala199Val.

Folding Structure	Mutation site	Mutation energy kcal/mol ^†^	Effect^‡^	van der Waals	**Electricity**	**Entropy**
primary	B-chain	0.46	neutral	0.34	0.27	0.19
	C-chain	-0.36	neutral	-0.92	0.23	-0.01
	D-chain	0.40	neutral	0.26	0.23	0.19
secondary	B, C-chain	-0.59	stabilizing	-2.85	0.84	0.51
	C, D-chain	-0.74	stabilizing	-2.67	0.88	0.19
	B, D-chain	0.52	destabilizing	-1.70	1.82	0.57
tertiary	B, C, D-chain	-1.80	stabilizing	-5.35	0.90	0.53

^†^ Differences between the folding free energy of mutated structure and wild-type protein. Sum of empirically weighted van der Waals force, electricity, entropy, and a non-polar surface dependent contribution (very small, not shown).

^‡^ A mutation effect was defined as neutral if the mutation energy was between -0.5 to 0.5 kcal/mol, stabilizing if the mutation energy was less than -0.5kcal/mol, and destabilizing if the mutation energies was greater than 0.5 kcal/mol.

## Conclusion

By comparing a selected population of brown Hanwoo with three closely related and unselected populations we identified 16 genomic regions suggesting recent selection in Hanwoo. These regions harbor 21 genes and 14 of these genes have been previously associated to production traits, especially meat traits and fat accumulation. Through the application of several analytical and comparative methods, we were able to single out the *APP* gene which was previously reported to be overexpressed in pigs with tender meat. A non-synonymous SNP (*Ala 199 Val*) that affected the protein structure was identified through sequencing and protein structure modelling. The most notable involvement of the *APP* gene is in its muscle degenerative function in humans; however, whether and how these human diseases and animal production traits are linked or caused through the same pathways and sequence variations remains unclear.

## Methods

### Ethics statement

The brown Hanwoo DNA was extracted either from AI bull semen straws or from blood samples obtained from different veterinary practitioners in the Hanwoo Improvement Center of the National Agricultural Cooperative Federation with the permission of the owners. The protocol was approved by the Committee on the Ethics of Animal Experiments of the National Institute of Animal Science (Permit Number: 2013–028). No ethics statement was required for the collection of DNA samples from the Brindle, Jeju Black Hanwoo, or Chinese Yanbian cattle because they were not sampled specifically for this study.

### Animals and genotype assays

The data on brown Hanwoo (N = 100) were collected from steers of candidate bulls for progeny testing in the Hanwoo Improvement Center of the National Agricultural Cooperative Federation in Seosan, Chungnam province, Korea. The DNA samples of the Brindle Hanwoo population (N = 20) were collected from steers from Hankyoung National University, Anseong and Ulleunggun Agriculture technology center, Gyeongsangbuk-do, Korea. The steers of Jeju Black (N = 20) Hanwoo were obtained from the subtropical animal experiment station of the National Institute of Animal Science (NIAS), Jeju island, Korea. Samples of Yanbian cattle (N = 39) were obtained from the Yanbian Agricultural College, Jilin in China. Angus data (N = 20) were obtained through the Animal Genetics and Breeding Unit, University of New England, Australia.

Genomic DNA for genotyping assays was extracted from semen or blood sample. The DNA was isolated from semen using the DNeasy 96 Blood and Tissue Kit (Qiagen, Valencia, CA, USA) with 100 μl of sperm added to 10 ml of Buffer 1. The mix was gently vortexed and centrifuged for 10 minutes at 4000 rmp to isolate pure cell pellets. Lysis of the cell walls was achieved by adding 300 μl Buffer 1 and 100 μl proteinase K to the pellet. The samples were incubated for 2 hours at 56°C. Purification of DNA was carried out with the DNeasy Blood & Tissue Kit; protocol 1 (Qiagen, Valencia, CA, USA) according to manufacturer’s protocol.

DNA quantification was performed using a NanoDrop 1000 (Thermo Fisher Scientific Inc., Wilmington, DE, USA). DNA samples were submitted for genotyping with total DNA of 900 ng, 260/280 ratio .1.8, and DNA concentration of 20 ng/ul. The single nucleotide polymorphism (SNP) genotyping was performed using the Illumina Bovine SNP 50K Bead chip (Illumina Inc., San Diego, CA). Approximately 200 ng of genomic DNA was used to genotype each sample on the chip. Samples were processed according to the Illumina Infinium-II assay. Normalized bead intensity data for each sample were processed with the Beadstudio 3.0 software (Illumina) which converted fluorescent intensities into SNP genotypes.

### Analysis of SNP statistics

Stringent filtering criteria were applied to the genotype data per breed. Briefly, SNPs were excluded from the analysis if they failed to provide genotypes in more than 5% of the cases, had median GC scores below 0.6, a GC scores under 0.6 in more than 90% of the samples, and deviated in heterozygosity by more than three standard deviations from the heterozygosity of other SNPs. Individual genotypes with GC scores under 0.6 were treated as missing. Loci out of Hardy-Weinberg equilibrium in a chi^2^-test for a cut-off p-value of 1^−15^, unmapped SNPs, SNPs on sex chromosomes, and SNPs with a minor allele frequency < 0.01 were also excluded. Finally, genotype data of 38,266 SNP remained after quality control.

### Analysis of genetic structure and relationship

Population structures were assessed with a principal component analysis (PCA) based on the genomic relationship matrix (GRM, **[56])**. Genotype records (0, 1, 2) were used to create a GRM. Through eigendecomposition, the principal components of the GRM were factorized. As such, a PCA summarizes the genetic similarity between subjects through eigenvalues and eigenvectors. The genetic variation within and between breeds were assessed with Weir and Cockerham’s *F*-statistics (*F*_*ST*_, **[19]**. *F*-statistics describe the reduction in heterozygosity in comparison to Hardy-Weinberg expectations. The *F*_*ST*_-statistic in particular describes the difference in allele frequencies between two independent populations with a potential value of 0 to 1, with 1 being the most different/ distantly related. *F*_*ST*_-distance matrices were used for hierarchical clustering using Ward’s minimum variance algorithm. European Angus samples were added as an out-group to the East-Asian populations.

To provide a finer quantification of the different ancestry proportions, we performed a model-based unsupervised hierarchical clustering of the individuals using Admixture 1.22 software **[57]**. Admixture provides a likelihood estimate of breed proportions dependent on allele frequencies of the assumed ancestral populations. Ancestral populations in an unsupervised analysis are clustered based on allele frequency similarities. We analyzed breed proportions with K = 2 to 4 assumed ancestral populations. Cross validation showed that K = 3 provided the best fit to our data.

### Detecting footprints of selection using Rsb score

Selection footprints per population were analyzed based on *extended haplotype homozygosities* (*EHH*) which is a measure for the breakdown of linkage disequilibrium with increasing distance from a SNP. Based on *EHH*, we computed *Rsb* scores according to Tang **[58]** using the *rehh* package in R **[59]**. *Rsb* is the standardized log-ratio of the integrated *EHHS* (*iES*) between pairs of populations and designed to detect sweeps that have occurred in only one population compared to another population. As our study is designed to detect sweeps specific to HW, genomic regions with extreme *Rsb* are indicative for signals of positive selection in HW. *Rsb* scores were transformed into *P*_*Rsb*_ = -log[Φ(*Rsb*)], assuming *Rsb* are normally distributed (under neutrality). *P*_*Rsb*_ can be interpreted as *log10(1 ⁄ P)* where *P* is the one-tailed hypothesis associated to the neutral hypothesis (no selection). To account for multiple testing, we applied the Benjamini-Hochberg false discovery rate correction (P-value = 0.05) **[21]**.

Haplotypes were predicted for a pooled sample (UN) from the three unselected breeds (BR, JB, and YB) to create a larger counter population to the selected HW and a more accurate haplotype prediction. Haplotypes of the HW and UN were predicted with fastPHASE **[60]**, respectively. We used default parameters except for the number of random starts for the EM-algorithm (-T option), which was set to 10 to reduce calculation time.

### Identification and annotation of candidate regions

Candidate regions with positive selection footprints were defined as containing at least two SNPs with a significant *Rsb* (FDR corrected P-value < 0.05) for a 1 Mb window (with a 0.5 Mb overlap) for the HW/UN. Candidate regions were further considered those containing at least two SNPs exceeding these thresholds for at least three population comparisons (**S3 Fig**). In case of several continuous windows with two SNPs or more, we combined the overlapping windows into one candidate region. Genes within the candidate regions were determined with the *intersectBed* command from the BedTools software **[61]**. The BedTools software allows to identify genes that overlap by at least one base out of a 4-bp integration site within a given candidate region. We also selected genes within +/- 300kb around SNPs that were identified in all four comparisons using “closetBed” in the BedTools. The genomic position of the genes was obtained from *'refGene'* information on the UCSC Genome Browser (http://genome.ucsc.edu/). To identify the functions/roles of genes in the candidate regions, we used the medScan database that provides sentences from MEDLINE abstracts [62]. QTL regions were identified from information on Cattle QTLs in the Animal QTLdb (Release 17; http://www.animalgenome.org/cgi-bin/QTLdb/BT/index). QTL locations by bp (Btau4.0) were downloaded and meat and production trait associated QTLs were selected. The associated names of these QTL types described in the Animal QTLdb were as follows: intramuscular fat, marbling score, average daily gain, yield grade, marbling score (EBV) shear force, tenderness score, body weight, height, feed conversion ratio, and average daily gain.

### Detection of sequence variation in the selection signals

We sequenced the entire genome of 10 HW and 10 YB cattle by randomly shearing 3 μg of genomic DNA to generate about 90bp inserts (Covaris INC, Woburn, USA). The fragmented DNA was amplified and end-repaired using T4 DNA polymerase, which has an inactive 5’-3’ exonuclease function. Illumina paired-end adaptor oligonucleotides were ligated to the sticky ends (Illumina Inc, San Diego, USA). We analyzed the ligation mixture by gel electrophoresis from which we purified fragments of 200–250bp length. These fragments were then sequenced on the HiSeq 2000 platform according to manufacturer’s specifications (Illumina Inc, San Diego, USA).

The sequences were aligned to the bovine reference genome (Btau4.0) using the Burrows-Wheeler Aligner (BWA; version 0.6.1) **[63]** with default parameters. SAMtools version 0.1.17 **[64]** was used for converting, sorting, and indexing the alignments. Duplicated reads were excluded from downstream analysis using Picard tools (Picard 2009, http://picard.sourceforge.net/). Local re-alignments and re-calibrations were performed with the Genome Analysis Toolkit framework (GATK; version 1.5.9, **[65]**. The initial novel SNP discovery was performed using the multi-sample SNP-calling procedure in the GATK package. All cut-off thresholds for filtering criteria, and cluster and window size were empirically derived and specific to this study (cluster Size = 3; clusterWindowSize = 10; MQ>-4; ((MQ0/1.0*DP))>0.1; QUAL<30; QD<5; Hrun>5; FS>200).

Additionally, we performed SNP annotation according to their functional type such as intergenic, 5’UTR, 3’UTR, coding SNP, or non-synonymous (nsSNP) in the target region using the ANNOVAR software **[66]**. The sequencing processes and more detailed information can be found in Choi et al. **[67].**

### Predicting protein structures

We used the PolyPhen program (Polymorphism Phenotype version 2.2; http://genetics.bwh.harvard.edu/pph2) to predict the effects of nsSNP (from the ANNOVAR analysis) on the protein structure. We based this analysis on the sequence data that we established in the previous section. This sequence data was translated into a protein sequence including the amino acid exchange of the nsSNP. PolyPhen provides a prediction about the effect of a SNP on the protein structure and categorizes them as *'benign'* (i.e. most likely lacking any phenotypic effect) or ‘*damaging’* (probably or possibly damaging: affecting protein function with higher or lower confidence, respectively) according a Bayesian approach. Regions with possibly or probably damaging nsSNP were further analyzed by investigating the haplotype structure surrounding these nsSNPs based on markers on the Illumina Bovine SNP 50K Bead chip. Haplotypes of the regions were generated with Haploview vs.4.2 **[68]** with the default algorithm described by Gabriel et al. 2002 **[69]**. Genes with damaging nsSNP were compared to the known genes located in our identified regions under selection.

Lastly, we predicted the bovine protein structure of the gene of interest (*APP*) through comparative homology modelling. We used the known human protein template structure (PDB code 3KTM) to explore mutation effects of the candidate genes due to a nsSNP. The homology modelling was performed using the MODELLER9v10 program within the Discovery Studio (DS) 3.5 molecular modelling packages (http://accelrys.com/products/discovery-studio/, Accelrys Inc, San Diego, USA). The detailed process is shown in **S4 Fig**.

## Supporting Information

S1 Fig The plot of genomic co-variance matrix based on the genotype data. The results showed that average relationship was 0.049, 0.05, 0.037 and 0.003 for Brindle, Jeju Black, Yanbian and Brown Hanwoo, respectively. Yellow means more genetically related between the individuals. (A) brown Hanwoo; (B) brindle Hanwoo; (C) Jeju black Hanwoo; (D) Chinese Yanbian.
(TIF)

S2 Fig Haplotype structure of candidate region around the bovine *APP* gene (BTA1:9–10.5Mb) in 4 East-Asian cattle breeds. Dark shading indicates strong linkage disequilibrium. (A) brown Hanwoo; (B) brindle Hanwoo; (C) Jeju black Hanwoo; (D) Chinese Yanbian.
(TIFF)

S3 Fig The *Rsb* plots of four cattle breed comparisons (HW/BR, HW/JB, HW/YB and HW/UN). HW: brown Hanwoo; BR: brindle Hanwoo; JB: Jeju black Hanwoo; YB: Chinese Yanbian cattle; UN: pooled unselected breeds.
(TIF)

S4 Fig The flowchart of prediction of protein structure. The first step is the use of PolyPhen program for predicting the functional effect of SNPs on the protein structure among the nsSNPs from resequencing data. The nsSNP (***Ala 199 Val***) of APP gene was analyzed the mutation effect based on the homology modeling using MODELLER program.
(TIF)

S1 Table Genomic regions with suggestive evidence (no FDR corrected) for recent positive selection in brown Hanwoo based on the *Rsb* statistic. (DOCX)
