# Supplementary figures and images for: Genomic Footprints in Selected and Unselected Beef Cattle Breeds in Korea
Source: PLoS One. 2016 Mar 29;11(3):e0151324. doi: 10.1371/journal.pone.0151324 (PMC4811422; doi:10.1371/journal.pone.0151324)

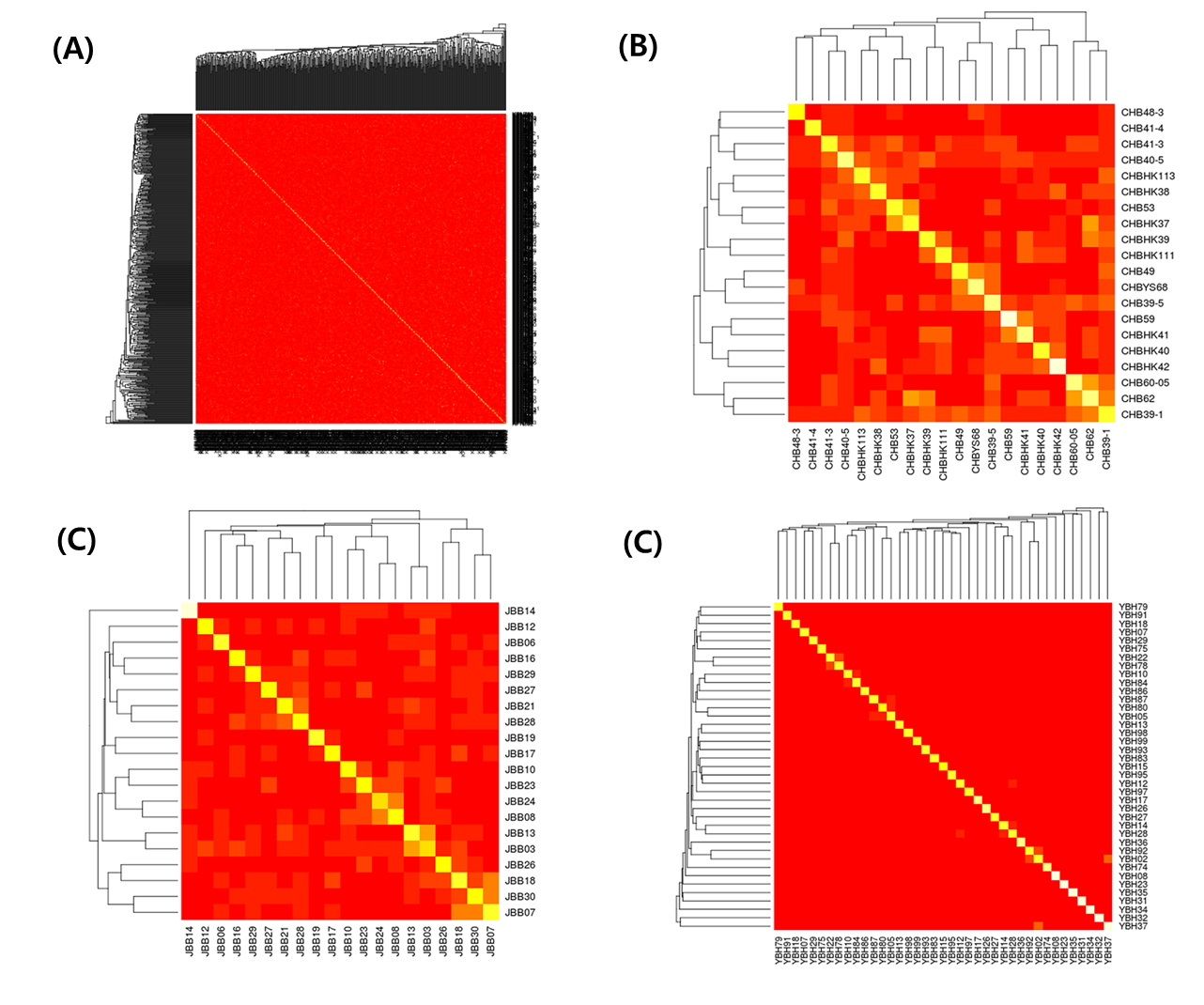

Supplement: S1 Fig — The results showed that average relationship was 0.049, 0.05, 0.037 and 0.003 for Brindle, Jeju Black, Yanbian and Brown Hanwoo, respectively. Yellow means more genetically related between the individuals. (A) brown Hanwoo; (B) brindle Hanwoo; (C) Jeju black Hanwoo; (D) Chinese Yanbian. (TIF) [file pone.0151324.s001.tif]

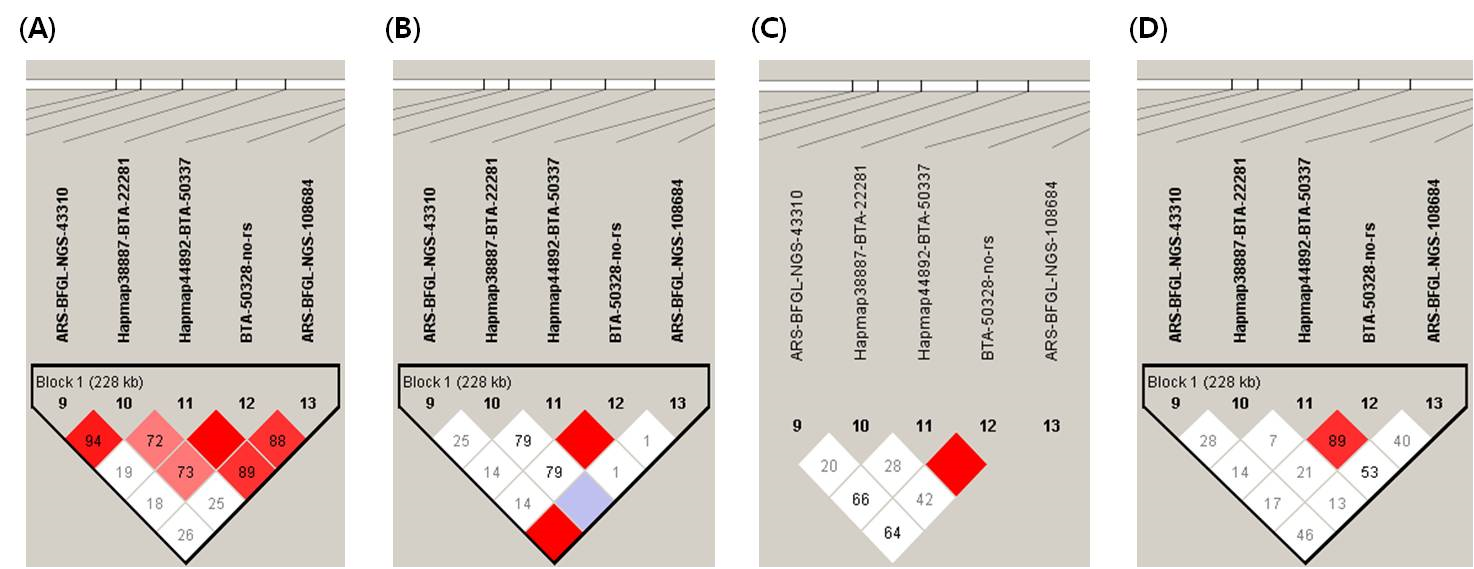

Supplement: S2 Fig — Dark shading indicates strong linkage disequilibrium. (A) brown Hanwoo; (B) brindle Hanwoo; (C) Jeju black Hanwoo; (D) Chinese Yanbian. (TIFF) [file pone.0151324.s002.tiff]

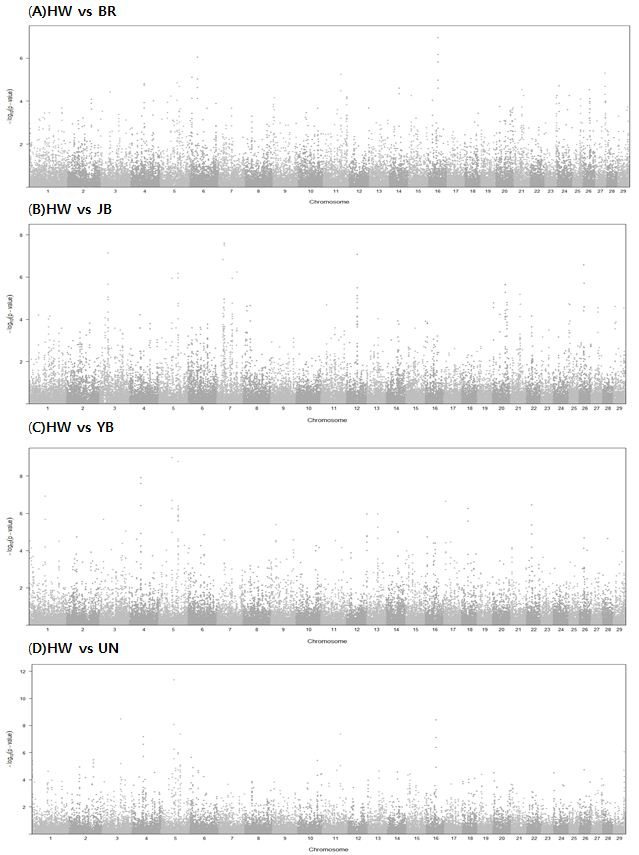

Supplement: S3 Fig — HW: brown Hanwoo; BR: brindle Hanwoo; JB: Jeju black Hanwoo; YB: Chinese Yanbian cattle; UN: pooled unselected breeds. (TIF) [file pone.0151324.s003.tif]

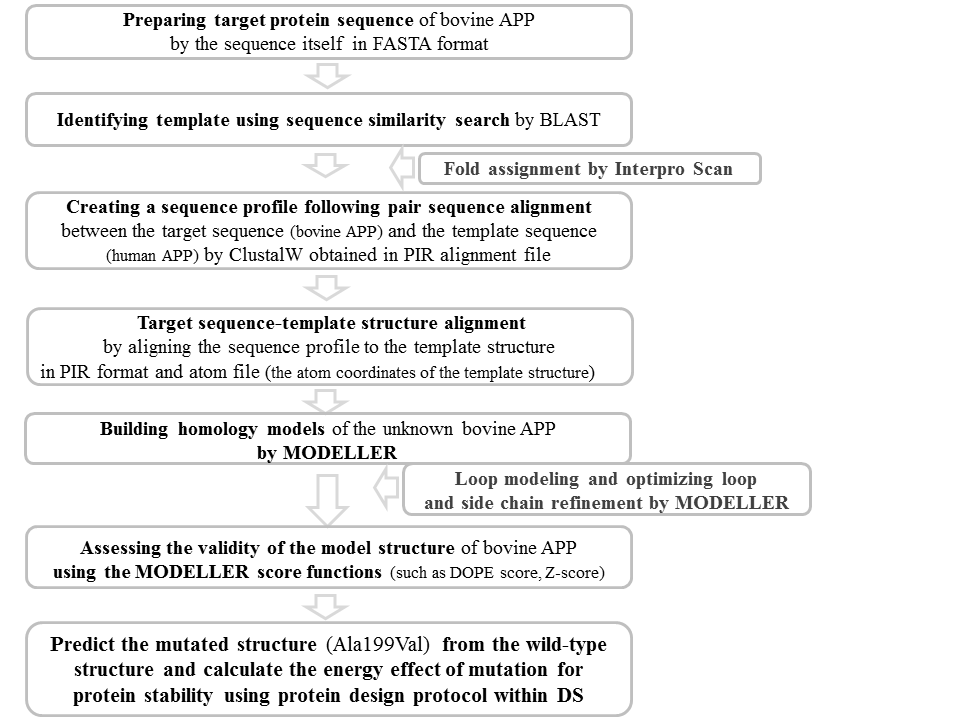

Supplement: S4 Fig — The first step is the use of PolyPhen program for predicting the functional effect of SNPs on the protein structure among the nsSNPs from resequencing data. The nsSNP (Ala 199 Val) of APP gene was analyzed the mutation effect based on the homology modeling using MODELLER program. (TIF) [file pone.0151324.s004.tif]
