# Supplementary material for: Genomic Footprints in Selected and Unselected Beef Cattle Breeds in Korea
Source: PLoS One. 2016 Mar 29;11(3):e0151324. doi: 10.1371/journal.pone.0151324 (PMC4811422; doi:10.1371/journal.pone.0151324)
Supplement: S1 Table — (DOCX) [file pone.0151324.s005.docx]

**S1 Table.** **Genomic regions with suggestive evidence (no FDR corrected) for recent positive selection in brown Hanwoo based on the *Rsb* statistic.**

| **Candidate regions**  **(Chr, Mb)** | | **Significant SNPs** | **Genes in: region** | **Trait associations** | **QTL information** |
| --- | --- | --- | --- | --- | --- |
| chr1 | 9-10.5 | UA-IFASA-5979, **Hapmap38887-BTA-22281*** | *APP*  *GABPA* | meat tenderness, obesity [32, 60, 61]  obesity [60] | Weaning weight-maternal milk |
| chr1 | 85-86.5 | **BTB-01702174***, BTB-01702150, Hapmap57884-rs29026697, BTB-01431374 |  | - | Marbling score, Longissimus muscle area, |
| chr1 | 128-129.5 | BTA-96197-no-rs, BTB-00056151, Hapmap50377-BTA-50302, BTB-00056188, BTA-50305-no-rs, BTB-00056214, BTB-00056233, ARS-BFGL-BAC-15540, BTA-50308-no-rs, BTB-01663737 | *TRPC1* | carcass and muscle cell growth [79] | - |
| chr2 | 102.5-104 | ARS-BFGL-NGS-99030, **ARS-BFGL-NGS-3990*, ARS-BFGL-NGS-107381***, **Hapmap34329-BES11_Contig247_1378***, **ARS-BFGL-NGS-67961***, ARS-BFGL-NGS-12630, ARS-BFGL-NGS-101219 | *ACADL MYL1* | fatty acids oxidation [62]  skeletal muscle differentiation [63] |  |
| chr3 | 1-2.5 | BFGL-NGS-117999, UA-IFASA-7054 | *RCSD1* | stress response [80] |  |
| chr3 | 95.5-97 | Hapmap30329-BTA-163245, ARS-BFGL-NGS-102021 | *PRKAA2* | fatty acids and intramuscular fat [81] | Body weight (birth) |
| chr4 | 47-48.5 | BTB-01562667, Hapmap55111-rs29019556, **BTB-01351245***, **BTB-01351221***, **ARS-BFGL-NGS-38285*, ARS-BFGL-NGS-108242***, ARS-BFGL-NGS-  79746, ARS-BFGL-NGS-23878, ARS-BFGL-NGS-835 | *RELN* | neural development [64] | Body weight, body energy content, Body length, length of productive life |
| chr4 | 58.5-60 | BTB-02035389, Hapmap39521-BTA-89033, BTB-01403737, Hapmap47769-BTA-89036, Hapmap25577-BTA-148332, ARS-BFGL-NGS-31435, BTA-70707-no-rs, BTA-70715-no-rs, BTB-00188171, BTA-70725-no-rs | *IMMP2L* | social interaction [82] | - |
| chr5 | 32.5-34 | BFGL-NGS-119788, Hapmap39286-BTA-73191, **ARS-USMARC-635*** | *WNT1*  *FAIM2* | coat color, melanocyte expansion [65]  obesity [66] | Body weight (slaughter, birth), Average daily gain, Subcutaneous fat increase |
| chr5 | 54.5-56 | ARS-BFGL-NGS-96600, **BFGL-NGS-111500*, Hapmap42656-BTA-73551*, BTA-73549-no-rs*, BFGL-NGS-111906***, Hapmap34023-BES4_Contig22  7_995, Hapmap34504-BES10_Contig662_1293, Hapmap49938-BTA-26588, Hapmap30892-BTA-162988, BFGL-NGS-119432, Hapmap43924-BTA-73528 | *AVPR1A* | mammalian social behaviour [67] | Body weight (slaughter, birth), body size, height |
| chr5 | 61-62.5 | A-IFASA-6196, Hapmap33935-BES11_Contig132_791, Hapmap46721-BTA-73566, Hapmap34521-**BES10_Contig695_990*, Hapmap34864-BES11_Contig325_836*, ARS-BFGL-NGS-44271***, ARS-BFGL-NGS-107167, Hapmap60668-rs29018280, Hapmap26410-BTA-143139, ARS-BFGL-NGS-12487 | *CD63 MYL6A, MYL6B* | skeletal muscle generation [68] | Body weight, pre-weaning average daily gain |
| chr6 | 10-11.5 | BTA-10940-no-rs, BTB-01322092, Hapmap32397-BTA-18899, BTB-01827197, **Hapmap42479-BTA-22862***, BTB-01879337, Hapmap52732-rs2  9023384, ARS-BFGL-NGS-99273, **BTA-88441-no-rs***, BTA-27165-no-rs | *NDST4* | fatness traits [69] | Body weight(birth, weaning, slaughter), Average daily gain |
| chr7 | 107-108.5 | BTA-80514-no-rs | *EFNA5* | lipid and phospholipid binding [83, 84] | - |
| chr8 | 28.5-30.5 | BTB-02079306, UA-IFASA-6308, ARS-BFGL-NGS-6044, BTA-93638-no-rs, ARS-BFGL-NGS-765, BTB-01270974, BTA-15966-no-rs, Hapmap50602-BTA-15967, BTB-01271264, Hapmap49891-BTA-15961, BTB-01271294, ARS-BFGL-NGS-23499, **BTB-01271445***, ARS-BFGL-NGS-100130 | *SH3GL2* | lipid binding and diabetis [70] | Average daily gain, residual feed intake, body weight |
| chr11 | 75.5-77 | ARS-BFGL-NGS-26350, ARS-BFGL-NGS-102717, **Hapmap35883-POMC_121F2-SNP1*, ARS-BFGL-NGS-39507***, BTA-104251-no-rs | *POMC* | obesity and coat color [71] | Body weight (mature, weaning) |
| chr12 | 84-85 | BTA-31480-no-rs, BFGL-NGS-112884 | *LAMP1* | co-localized in *TYRP1* (brown coat color) [85, 86] | - |
| chr13 | 45.5-47 | BTB-00525974, ARS-BFGL-NGS-24766, **BFGL-NGS-116062*,** ARS-BFGL-NGS-810, **BFGL-NGS-116977***, ARS-BFGL-NGS-20698 | *ADRB2* | body mass index and adipogenesis [72] | Feed conversion ratio |
| chr13 | 66.5-68 | ARS-BFGL-NGS-32791, ARS-BFGL-NGS-3823 | *GHRH* | carcass trait and eye muscle area [87] | - |
| chr14 | 41.5-43 | **ARS-BFGL-NGS-59210*, BTB-01709715***, ARS-BFGL-NGS-5105 | *FABP4, FABP5* | intramuscular fat [73] | Average daily gain, body weight(birth) |
| chr15 | 12.5-14.5 | Hapmap59061-rs29016275, Hapmap59332-rs29016542, UA-IFASA-2285, ARS-BFGL-NGS-10627, ARS-BFGL-BAC-31586, ARS-BFGL-NGS-10723, **Hapmap35322-BES8_Contig457_1759*, ARS-BFGL-NGS-17747***, Hapmap41817-BTA-29310, ARS-BFGL-NGS-17568, Hapmap35448-SCAFFOLD52197_3147, ARS-BFGL-NGS-25540 | *MTMR2 CWC15* | mammary gland metabolism [74]  reproduction [75] | Body weight(birth, yearling), semen volume |
| chr15 | 39.5-40.5 | BFGL-NGS-119103, Hapmap50841-BTA-99608, BTB-00597065, ARS-BFGL-NGS-73907, ARS-BFGL-NGS-76827 | *USP47* | cell growth and survival [88] | Height (mature) |
| chr16 | 0-1.5 | BTB-00623605, BTB-00623664, BTB-00623624, Hapmap51407-BTA-121049, BTB-00623817, Hapmap47596-BTA-38603 | *ATP2B4* | mammary gland [89] | Body weight(weaning), body depth |
| chr16 | 8-9 | Hapmap54310-rs29012181, Hapmap49982-BTA-39672, BTA-24867-no-rs |  | - | Body weight(slaughter, weaning) |
| chr16 | 34-35 | ARS-BFGL-NGS-34764, ARS-BFGL-NGS-25955, BTA-38752-no-rs, ARS-BFGL-NGS-56401, BTB-00634111 | *ATP1B1* | feed efficiency and carcass weight [90, 91] | Body weight (weaning, birth), average daily gain |
| chr18 | 25.5-27 | ARS-BFGL-NGS-20779, **UA-IFASA-4931*, ARS-BFGL-BAC-34198***, ARS-BFGL-NGS-91084, BTA-42834-no-rs |  | - | Resigual feed intake, body weight (weaning) |
| chr19 | 15-16.5 | BTB-00738813, Hapmap53903-rs29027471, ARS-BFGL-NGS-101716, **UA-IFASA-8305***, ARS-BFGL-NGS-26149, Hapmap53971-rs29017666 | *ACCN1* | QTL for reproduction [76] | Calf size, body weight (mature, yearling), residual feed intake |
| chr20 | 69.5-71 | BTA-51172-no-rs, ARS-BFGL-NGS-44059, ARS-BFGL-NGS-28789, ARS-BFGL-NGS-103819, BTA-51187-no-rs, ARS-BFGL-NGS-15256 | *SRD5A1* | steroid hormone biosynthesis | - |
| chr22 | 21.5-23 | ARS-BFGL-NGS-88777, UA-IFASA-6291, BTB-00841829, UA-IFASA-6407 | *BHLHE40* | fatty acids regulation in muscle [34] | Body weight (yearling) |
| chr22 | 25-26.5 | ARS-BFGL-NGS-44075, BTA-53914-no-rs, ARS-BFGL-NGS-109363 | *CNTN6* | brain development [92] | Body weight (yearling), height (mature) |
| chr26 | 2.5-4 | BTA-61213-no-rs, ARS-BFGL-NGS-46925, Hapmap58711-rs29020973, BTB-00920094 |  | - | Body weight (yearling), height (mature), retail product yield |
| chr29 | 44.5-47 | ARS-BFGL-NGS-59421, **ARS-BFGL-NGS-21416, CAPN1_1***, UA-IFASA-1370, UA-IFASA-9734, ARS-BFGL-NGS-19057, ARS-BFGL-NGS-82048, UA-IFASA-5680, **BTA-66033-no-rs***, **ARS-BFGL-NGS-74975***, **ARS-BFGL-NGS-20927***, **ARS-BFGL-NGS-34609***, ARS-BFGL-NGS-104441, Hapmap30072-BTA-65952, ARS-BFGL-NGS-29040 | *CAPN1* | carcass trait and meat tenderness [77, 78] | Body capacity, tenderness score, shear force, resudial feed intake |

***** SNPs with a significant FDR corrected P-values (< 0.05).
